# Cognitive reflection test: The effects of the items sequence on scores and response time

**DOI:** 10.1371/journal.pone.0279982

**Published:** 2023-01-10

**Authors:** Inmaculada Otero, Pamela Alonso

**Affiliations:** Faculty of Labour Relations, Department of Political Science and Sociology, University of Santiago de Compostela, Santiago de Compostela, Spain; Universitat Jaume I Departament d’Economia, SPAIN

## Abstract

This paper aims to expand the literature on the determinants of the Cognitive Reflection Test scores, exploring the effects that the items sequence has on (1) Cognitive Reflection Test scores, (2) response time, (3) the relationship between Cognitive Reflection Test scores and response time, and (4) Cognitive Reflection scores, response time, and the relationship between both variables on men and women. The current study also explored the sex differences on Cognitive Reflection Test and response time according to items sequence. The results showed that manipulating the items sequence, the performance on the Cognitive Reflection Test improved significantly, but the response time were not significantly affected, although the results suggest that first items of the sequence could be working as training items. A positive relationship between Cognitive Reflection Test scores and response time was also found, except when the scores were maximized. Finally, some differences between men and women on the results were also found. The implications of these findings are discussed.

## Introduction

Dual-Process Theories assume that human reasoning operates on the base of two types of cognitive processes: one fast and intuitive and another slow and reflective. Researchers have referred to them in multiple ways, heuristic vs. analytic [1–3], automatic vs. algorithmic [4], associative system vs. rule-based system [5], and system 1 vs. system 2 [6,7], among others; but currently the terminology more recommended is Type 1 (T1) and Type 2 (T2) thinking [8–10].

T1 thinking produces quick, emotional, intuitive, impulsive as well as associative judgments. It works effortless, automatizing behaviors by means of learning and the consistent experience with the environment. For this reason, the two main functions of T1 thinking are to carry out routine activities which were automatized [4,11], and to perceive and codify the information from environment to facilitate the T2 thinking [1,12].

T2 thinking produces reflective, rational, deliberative as well as rule-guided judgments. It works slow, with effort and concentration, and demands cognitive load [6]. The main function of T2 thinking is to control the T1 processing, overriding its responses when they are erroneous or not appropriate [5,6,13–16]. It is also responsible for operating when T1 thinking cannot activate, for instance, due to the lack of previous experience dealing new situations [3,6].

People vary in their inclination to spontaneously engage one of these types of cognitive processes, and this individual difference is labelled cognitive reflection. Kahneman and Frederick [6,17] defined the cognitive reflection (CR) as the ability or disposition to annulate the first impulsive response that our mind offers (T1 thinking), and to activate the reflection mechanisms (T2 thinking) that allows us to find a response, to make a decision, or to carry out a specific behavior in a more thoughtful way.

Kahneman and Frederick [6,17,18] also developed the well-known 3-item Cognitive Reflection Test (CRT) to assess the inclination to spontaneously engage one of the two types of processing. The items are three simple arithmetical problems that trigger an immediate and apparently correct answer. To solve them, individuals have to (1) suppress this immediate answer of T1 thinking and (2) to switch the T2 thinking for deliberately finding the correct answer [19,20]. According to the above, higher scores on CRT indicate the propensity to activate T2 thinking spontaneously, and lower scores indicate the tendency to activate T1 thinking.

Previous studies have shown that a high percentage of people fail to answer the three items of CRT correctly and, consequently, more people tend to activate by default the T1 thinking instead of T2. For instance, Frederick [17] found that the 63% of participants solved 0 or 1 items correctly and only the 17% answered the three items right. In the meta-analysis of Brañas-Garza et al. [21], results reported that the 60.72% of participants solved 0 or 1 items right and only 18.17% provided all answers correctly. These findings have incremented the interest for exploring why many people solve wrong the CRT items, i.e., what determines the inclination of people to activate spontaneously the T1 or the T2 thinking performing the test.

Therefore, the current study aims to contribute to the literature on the determinants of CRT scores by exploring the effects that the items sequence (i.e., the order of administration the CRT items) have on (1) CRT scores; (2) CRT response time; (3) the relationship between CRT score and response time; and (4) CRT scores, response time, and the relationship between both variables on men and women. Additionally, this study also aims to explore the sex differences in CRT scores and response time according to the items sequence.

### Literature on the determinants of CRT scores

The CR literature have suggested that the CRT scores might be determined by several variables, regarding to individual differences (e.g., cognitive abilities, the tendency to use heuristics and cognitive biases, and the pragmatic competence), to features of the test (e.g., the rhetorical structure of items, the numerals that items involve, and the items sequence), and regarding to features of the context where CRT is performing (e.g., the format that CRT is administrated [computer vs. paper and pencil], the moment of the day that CRT is answered, and the cognitive load that people are exposed).

The variables of individual differences were most deeply explored. In this sense, the cognitive abilities were suggested as a relevant determinant. Several studies have evidenced a relationship between CRT scores and cognitive abilities, such as, general mental ability [16,17,22–24], numerical ability [25–30], verbal ability [31–35], mechanical-spatial ability [35–37], and other cognitive variables as working memory capacity [24,25,38], executive functions [22,39], and perceptual speed [31,40]. However, CRT scores are more robust related to general mental ability and numerical ability. According to a recent meta-analysis conducted by Otero et al. [41], the best estimation of the relationship between CR and general mental ability and numerical ability is .53 and .54, respectively. These findings suggest that people score high on cognitive abilities also score high on CRT.

Another individual difference that might determine the CRT scores is the tendency to use heuristics elaborating responses. Kahneman and Frederick [6] assert that people might be answering the CRT items intuitively using the attribute substitution bias. This bias is used when people are confronted with a difficult question. Without being conscious, individuals substitute the difficult question by other easier in order to reduce the cognitive load of thinking. For instance, the attribute substitution bias in the item *“A bat and a ball cost $1*.*10 in total*. *The bat costs $1*.*00 more than the ball*. *How much does the ball cost*?*”* consists in replace the critical relation *“The bat costs $1*.*00 more than the ball”* by an absolute affirmation that is simpler, i.e., *“the bat cost $1*.*00”*. Thus, people response with the intuitive answer instead of the correct one [42,43].

The pragmatic competence is another individual difference that was suggested as determinant of CRT scores [44]. The pragmatic competence is the capacity to decontextualize and depersonalize the information of the items to avoid answering the immediate answer. Macchi and Bagassi [44] assert that the lack of pragmatic competence is due to the item which is interpreted based on the context and not abstracting from it. The rhetorical structure of the items becomes the text ambiguate, and trigger people to use the attribution substitution bias to find an answer. According to authors, the pragmatic competence facilitates the disambiguation of the statement and the activation of T2 thinking.

Parallel, Liberali et al. [45] hold that the rhetorical structure of statements triggers the illusion that the answer of the items is given in the own statement. They suggest that the failure solving the CRT items might be due to thinking superficially on items. Thus, people would be answering the using the words of the statement without understanding its meaning. For example, many people realise that the correct answer of the item “*if it takes 5 machines 5 minutes to make 5 widgets*, *how long would it take 100 machines to make 100 widgets*?” is 100, because the answer is immediately suggested by the statement. If 5 machines make 5 widgets in 5 minutes, then 100 machines must make 100 widgets in 100 minutes. This fact suggests that people answering intuitively do not check their responses [14,17,45].

The response time has also been suggested as determinant of the individual differences on CRT scores. Preceding studies have shown that intuitive answers are more related to fast responses than rational answers (see, for instance [46–48]). Böckenholt [49] found that the intuitive answers of CRT items were given significantly faster than the deliberate answers. Kinnunen and Windmann [50] and Mata and Almeida [51] found that people are more inclined to give the intuitive response when they are instructed to answer fast on the CRT than when they are encouraged to answer slowly. These findings also support the idea that the failure solving the CRT items might be due to thinking quickly and superficially.

According to the above, the rhetorical structure of items might be a determinant on the CRT scores related to the test´s features. Previous studies have shown that reformulating the items statements, subjects find them easier to grasp the correct response. For instance, Macchi and Bagassi [44] found that the 90% of participants correctly answered the bat-and-ball item when the price of the bat and the ball was asking separately, against the 10% for the original version. Mata et al. [52] found that subjects who detected some critical changes in the redaction of items after to response the original ones showed more tendency to correctly answer the test. Finally, De Neys and colleagues [42,53,54] found that people answered significantly more CRT items right in the “non-conflict” version than in the original one. In the “non-conflict” version, the CRT items are worded to not trigger an immediately answer (for example, “*A bat and a ball cost $1*.*10 in total*. *The bat costs $1*.*00*. *How much does the ball cost*?”).

Another determinant related to features of the test was suggested by Mastrogiorgio and Petracca [55]. They propound that numerals (i.e., the numerical symbols) that items involve might determine the CRT scores. This affirmation is based on the idea that some numerals invite more computation than other, and consequently people are more propensity to activate the T2 thinking. To understand this idea, they differentiate between numbers (i.e., expression of magnitudes) and numerals (i.e., numerical symbols). In the study, Mastrogiorgio and Petracca [55] administrated two versions of the bat-and-ball item. The items were (1) algebraically and arithmetically homogeneous, i.e., both items involve the same mathematical procedure or calculation and similar numbers (i.e., magnitudes but not numerals); and (2) identically worded to control the effects of the rhetorical structure. The results showed that 56.70% of participants correctly answered the original item vs. 80% for the isomorphic version. Hence, Mastrogiorgio and Petracca [55] assert that the numerals, instead of numbers and the wording of the statement, might explain the individual differences on the CRT scores. The items that involve less prominent numbers might demand more cognitive load than the same items that involve prominent numbers [e.g., 10,15,20], forcing the activation of the T2 thinking in a greater extend.

In addition, Brañas-Garza et al. [21] suggested the sequence of CRT items as another determinant on the CRT scores. The meta-analyses developed by Brañas-Garza et al. [21] showed that participants scored higher on CRT when the items were administrated in the original order (i.e., bat-and-ball item, followed by widgets item, and the lily-pads item) than when they were administrated in whatever other combination. Hence, the CRT scores could be determined manipulating the order that items are administrated.

Other variables related to features of the CRT that have also been explored were the font (i.e., fluent vs. disfluent font; see, for instance [56,57], and the language (i.e., native vs. foreign language; see [58]) that items are wording, but in both cases, the results were not conclusive.

Finally, variables regarding to features of the context where CRT is performing has also been suggested as determinants of CRT scores. For example, Brañas-Garza et al. [21] assert that the format that the CRT is administrated might affect the scores on the test. They observed that people scored higher on the CRT when the test was administrated on computer than when it was administrated on paper and pencil. Böckenholt [49] found that the moment of the day that the CRT is answered might affect to scores. He observed that people answered significantly more items correctly when the CRT was answered in the morning than in the afternoon. Lastly, Johnson et al. [54] and Morsanyi et al. [59] suggested that the cognitive load that people are exposed answering the test could affect the CRT scores. They observed that people who were exposed to cognitive load scored lower than people who were not exposed to cognitive load.

### Aims of the study

To sum up, CR literature have shown evidence that several variables, concerning to individual differences, features of the test, and features of the context, could determine the CRT scores. However, these findings are scarce and insufficient, and new studies would be appropriate. Hence, the purpose of this study is to explore the effects that items sequence has on (1) CRT scores; (2) CRT response time; (3) the relationship between CRT score and response time; and (4) the CRT scores, response time, and the relationship between both variables regarding the sex. In addition, the current study also aims to determine the sex differences in CRT scores and response time according to items sequence.

## Method

### Participants

The sample was composed of 602 adults of a Western European country. The 63.00% of the sample were woman (*n* = 379) and the average age was 21.39 (*SD* = 3.83, range = 18–50).

### Procedure and instruments

Small group sessions were organized to conduct the experiment. Participation was voluntary and subjects received an economic bonus in return. All the participants provided their written and informed consent to take part in the study. Given the fact that the data provided by the subjects were confidential, that their treatment was anonymous at the variable level, and that they were exclusively used for the current study, this study was exempt from the need of approval from Bioethics Committee of the University of Santiago de Compostela.

A measure of 13-items was administrated as CRT (henceforth CRT-13). This compound was created from CRT-3 of Frederick [16,17] and CRT-10 of Salgado [60]. Two alternatives of answers were disposed per item, one representing the intuitive answer of T1 thinking and another representing the reflective answer of T2 thinking. Participants must choose one of them and they did not have a limited time. The scores could range from 0 (no items answering correctly) to 13 (all items answering correctly). Thus, higher scores indicate greater CR. The omega´s reliability coefficients for this sample were .82, .76, and .78 for CRT-13, CRT-3, and CRT-10, respectively. The test-retest reliability coefficients were .78 for CRT-13, .71 for CRT-3, and .72 for CRT-10.

The CRT-13 was administered on computer, using the Millisecond Inquisit 5 Lab software [61] to record time reaction. Each item was displayed individually in the center of screen and the answer options was displayed immediately below in a parallel form. The “next” button, located at the right bottom margin of the screen, had to be pressed to move forward in the test. The prior items had to be completed to continue answering the remaining, and changes in previous responses were not allowed. These instructions were notified to participants before starting the experiment.

The sequence of CRT items was manipulated in the experiment. Three experimental conditions were designed according to the position of CRT-3 items into CRT-10 (i.e., at the beginning, in the middle, and at the end of the sequence). Hence, condition 1, the CRT-3 was administrated followed by CRT-10. Condition 2, the first six items of CRT-10 were disposed followed by the CRT-3, and the last four items of CRT-10. Finally, condition 3, the CRT-10 was administrated followed by CRT-3. Table 1 shows the frequencies and percentages of participants for experimental conditions. Participants were assigned at random to one of these experimental conditions.

**Table 1:** Sample distribution for experimental conditions.

	Condition 1	Condition 2	Condition 3	Total
Total Sample	197 (32.7%)	199 (33.1%)	206 (34.2%)	602 (100%)
Men	69 (35.0%)	68 (34.2%)	86 (41.7%)	223 (37.0%)
Women	128 (65.0%)	131 (65.8%)	120 (58.3%)	379 (63.0%)

## Results

### The effects of items sequence on CRT scores

Table 2 shows the results of the effects that items sequence had on CRT scores. From top to bottom, table reports data from the total sample and from men and women, considered separately, regarding to CRT-13, CRT-3, and CRT-10. From left to right, table reports the mean and the standard deviation of CRT scores from experimental conditions and the differences between experimental conditions in the CRT performance, using the statistic of Fisher´s *F* and the effect size of Dunlap´s *d* [62].

**Table 2:** Mean, standard deviations, and differences in CRT scores according to experimental conditions.

	CRT scores						Differences in CRT scores					
	Condition 1		Condition 2		Condition 3		Condition 1 vs. 2		Condition 1 vs. 3		Condition 2 vs. 3	
	$\bar{X}$	*SD*	$\bar{X}$	*SD*	$\bar{X}$	*SD*	*d*	*F*	*d*	*F*	*d*	*F*
*Total Sample*												
CRT-13	7.22	3.39	7.90	3.01	7.19	3.11	-.21	4.48*	.01	.01	.23	5.45*
CRT-3	1.27	1.12	1.77	1.02	1.50	1.01	-.47	21.10***	-.22	4.53*	.27	7.07**
CRT-10	5.94	2.55	6.13	2.25	5.69	2.41	-.08	.60	.10	1.07	.19	3.64
*Men*												
CRT-13	8.48	3.34	9.24	2.73	8.38	2.90	-.25	2.11	.03	.04	.30	3.46
CRT-3	1.57	1.11	2.13	0.96	1.79	1.02	-.54	10.27**	-.21	1.74	.34	4.49*
CRT-10	6.91	2.48	7.10	2.02	6.59	2.21	-.08	.24	.14	.72	.24	2.18
*Women*												
CRT-13	6.54	3.22	7.21	2.93	6.33	2.98	-.22	3.04*	.07	.27	.30	5.48*
CRT-3	1.12	1.10	1.58	1.01	1.29	.96	-.44	12.51***	-.16	1.77	.29	5.39*
CRT-10	5.42	2.44	5.63	2.20	5.04	2.34	-.09	.50	.16	1.57	.26	4.17*

*Note*. Condition 1 = CRT-3 at the beginning of the sequence; Condition 2 = CRT-3 in the middle of the CRT-10; Condition 3 = CRT-3 at the end of the sequence; $\bar{X}$ = mean; *SD* = standard deviation; CRT-13 = compound of CRT-3 [17] and CRT-10 [60].

*p ≤ .05

**p ≤ .01

***p ≤ .001.

As it can be seen, the results show that participants scored significantly higher on CRT-13 and on CRT-3 when Frederick´s items were administrated in the middle of CRT-10 (condition 2) than when they were administrated in either of the two sequencies (at the beginning *d* = -.21 for CRT-13 and *d* = -.47 for CRT-3; and at the end *d* = .23 and *d* = .27, respectively). The effect sizes were more robust for CRT-3 than for CRT-13. Results also show that participants scored higher on CRT-3 when they were administrated at the end of CRT-10 (condition 3) than when they were administrated at the beginning of the test (condition 1, *d* = -.22). However, no differences were found on CRT-10 across the conditions. Despite the significant differences, the effect sizes finding for the total sample were mainly small or very small, with a couple of exceptions, showing a moderate effect size.

Regarding men and women, results show that both groups scored higher on CRT-3 when these items were in the middle of CRT-10 (condition 2) than when they were in either one of two sequencies (at the beginning *d*_M_ = -.54 and *d*_W_ = -.44; and at the end *d*_M_ = .34 and *d*_W_ = .29). In the case of women, results also show that items sequence had some effects on CRT-13 and CRT-10 scores. For instance, women scored higher on CRT-13 when CRT-3 items were administrated in the middle of CRT-10 than when they were administrated in remained sequences (*d* = -.22 in condition 1 vs. 2, and *d* = .30 in condition 2 vs. 3). Finally, the women´s scores on CRT-10 were statistically higher when the CRT-3 items were administrated in the middle of the test than when they were administrated at the end (*d* = .26). However, men´s scores on the CRT-10 and on the CRT-13 were not affected by the items sequence. Again, despite the significant differences, the effect sizes finding for men and women samples were mainly small or very small, with a couple of exceptions.

#### Sex differences in CRT scores

Table 3 shows sex differences on CRT scores for experimental conditions. As it can be seen, men scored higher on CRT-13, CRT-3, and CRT-10 than women in all experimental conditions. These differences were moderated [63] and statistically significant. The effect sizes ranged from .41 to .61 in condition 1, from .55 to .71 in condition 2, and from .51 to .70 in condition 3. The differences were more robust in CRT-13 than in CRT-3 and CRT-10, suggesting that the number of items could moderate the sex differences in scores.

**Table 3:** Sex differences in CRT scores according to experimental conditions.

	Condition 1						Condition 2						Condition 3					
	Men		Women				Men		Women				Men		Women			
	$\bar{X}$	*SD*	$\bar{X}$	*SD*	*d*	*F*	$\bar{X}$	*SD*	$\bar{X}$	*SD*	*d*	*F*	$\bar{X}$	*SD*	$\bar{X}$	*SD*	*d*	*F*
CRT-13	8.48	3.34	6.54	3.22	.60	15.81***	9.24	2.73	7.21	2.93	.71	22.53***	8.38	2.90	6.33	2.98	.70	24.32***
CRT-3	1.57	1.11	1.12	1.10	.41	7.43**	2.13	.96	1.58	1.01	.55	13.88***	1.79	1.02	1.29	.96	.51	12.92***
CRT-10	6.91	2.48	5.42	2.44	.61	16.61***	7.10	2.02	5.63	2.20	.69	21.35***	6.59	2.21	5.04	2.34	.68	23.08***

*Note*. Condition 1 = CRT-3 at the beginning of the sequence; Condition 2 = CRT-3 in the middle of the CRT-10; Condition 3 = CRT-3 at the end of the sequence; $\bar{X}$ = mean; *SD* = standard deviation; CRT-13 = compound of CRT-3 [17] and CRT-10 [60].

^ƚ^*p* < .10

*p ≤ .05

**p ≤ .01

***p ≤ .001.

Finally, although the sex differences in CRT in condition 2 were higher than in condition 1 and 3, the differences between effect sizes were not statistically significant (range of *z* = .05 to .54). These findings indicate that items sequence could have no effects on sex differences on CRT scores.

### The effects of items sequence on response time

Table 4 shows the effects that the items sequence had on the time that participants spent responding to the CR tests.

**Table 4:** Mean, standard deviations, and differences in CRT response time (in seconds) according to experimental conditions.

	CRT Response Time						Differences in Response Time					
	Condition 1		Condition 2		Condition 3		Condition 1 vs. 2		Condition 1 vs. 3		Condition 2 vs. 3	
	$\bar{X}$	*SD*	$\bar{X}$	*SD*	$\bar{X}$	*SD*	*d*	*F*	*d*	*F*	*d*	*F*
*Total Sample*												
CRT-13	646.01	298.93	662.06	272.64	693.79	294.55	-.06	.31	-.16	2.61	-.11	1.26
CRT-3	111.56	59.32	101.33	45.70	102.92	58.31	.19	3.70 ^ƚ^	.15	2.17	-.03	.09
CRT-10	534.45	261.98	560.72	244.10	590.87	254.50	-.10	1.07	-.22	4.81*	-.12	1.48
*Men*												
CRT-13	667.44	284.88	674.23	249.15	724.35	321.43	-.03	.02	-.19	1.33	-.17	1.12
CRT-3	122.32	71.41	100.70	44.10	105.55	62.52	.36	4.53*	.25	2.43	-.09	.29
CRT-10	545.11	237.28	573.53	220.43	618.79	273.85	-.12	.53	-.29	3.12^ƚ^	-.18	1.23
*Women*												
CRT-13	634.46	306.70	655.74	284.78	671.90	272.96	-.07	.34	-.13	1.03	-.06	.21
CRT-3	105.76	51.02	101.66	46.67	101.03	55.29	.08	.45	.09	.49	.01	.01
CRT-10	528.71	275.10	554.08	256.09	570.86	238.83	-.10	.59	-.16	1.65	-.07	.29

*Note*. Condition 1 = CRT-3 at the beginning of the sequence; Condition 2 = CRT-3 in the middle of the CRT-10; Condition 3 = CRT-3 at the end of the sequence; $\bar{X}$ = mean; *SD* = standard deviation; CRT-13 = compound of CRT-3 [17] and CRT-10 [60].

^ƚ^*p* < .10

*p ≤ .05

**p ≤ .01

***p ≤ .001.

The results show no statistically significant differences between experimental conditions in the RT of CRT-13, but they show small differences in the RT of CRT-3 and CRT-10. Participants used more time responding the CRT-10 when the CRT-3 items were administrated at the end of the sequence (condition 3) than when they were administrated at the beginning (condition 1, *d* = -.22). Moreover, participants spent marginally more time answering the CRT-3 when these items were administrated at the beginning of the sequence (condition 1) than when they were administrated in the middle (condition 2, *d* = .19) but, in both cases, small differences were found [63].

Regarding sex, no statistically significant differences were found in the RT for women (range of *d* = .01 to .16). However, men spent more time answering the CRT-3 when these items were administrated at the beginning of the sequence (condition 1) than when they were administrated in the middle (condition 2, *d* = .36). Likewise, men used more time deliberating about CRT-10 when the CRT-3 items were located at the end of the sequence (condition 3) than when they were located at the beginning (condition 1, *d* = -.29). The findings indicate that the sequence of items could have effects on the time that men spend responding the tests, but not on the time that women use to answering.

#### Sex differences in response time

Table 5 shows the sex differences in RT for experimental conditions. As it can be seen, results show no sex differences in RT. It has just found that men spent more time answering the CRT-3 items than women when these items were located at the beginning of the sequence (condition 1). However, the differences were small and marginally significant (*d* = .28). No sex differences were found in conditions 2 and 3.

**Table 5:** Sex differences in CRT response time (in seconds) according to experimental conditions.

	Condition 1						Condition 2						Condition 3					
	Men		Women				Men		Women				Men		Women			
	$\bar{X}$	*SD*	$\bar{X}$	*SD*	*d*	*F*	$\bar{X}$	*SD*	$\bar{X}$	*SD*	*d*	*F*	$\bar{X}$	*SD*	$\bar{X}$	*SD*	*d*	*F*
CRT-13	667.44	284.88	634.46	306.70	.11	.54	674.23	249.15	655.74	284.78	.07	.21	724.35	321.43	671.90	272.96	.18	1.59
CRT-3	122.32	71.41	105.76	51.02	.28	3.54^ƚ^	100.70	44.10	101.66	46.67	-.02	.02	105.55	62.52	101.03	55.29	.08	.300
CRT-10	545.11	237.28	528.71	275.10	.06	.18	573.53	220.43	554.08	256.09	.08	.28	618.79	273.85	570.86	238.83	.19	1.78

*Note*. Condition 1 = CRT-3 at the beginning of the sequence; Condition 2 = CRT-3 in the middle of the CRT-10; Condition 3 = CRT-3 at the end of the sequence; $\bar{X}$ = mean; *SD* = standard deviation; CRT-13 = compound of CRT-3 [17] and CRT-10 [60].

^ƚ^ *p* ≤ .10

*p ≤ .05

**p ≤ .01.

### The effects of items sequence on the relationship between CRT scores and response time

Another purpose of this study is to explore the effects that items sequence has on the relationship between CRT scores and RT. The observed and the corrected correlations between CRT scores and RT were estimated for conditions. We calculated the internal consistency reliability of RT and CR measures to correct the observed correlations by measurement error. The magnitude of the reliabilities appears in Table 6.

**Table 6:** Internal consistency reliability of response time and CR tests.

	Total Sample	Men	Women
*Response Time*			
RT-13	.79	.78	.79
RT-3	.49	.54	.45
RT-10	.75	.73	.76
*Cognitive Reflection*			
CRT-13	.82	.82	.80
CRT-3	.76	.76	.75
CRT-10	.78	.78	.75

*Note*. The magnitude of reliabilities for CR tests is omega coefficient. RT-13 = response time of CRT-13; RT-10 = response time of CRT-10; RT-3 = response time of CRT-3; CRT-13 = compound of CRT-3 [17] and CRT-10 [60].

Table 7 shows the correlations between the scores of CR test and its correspondence RT value for experimental conditions. The results show positive and statistically significant correlations between variables when CRT-3 items were administrated at the beginning (condition 1) and at the end of the test (condition 3). The magnitude of the observed correlations ranged from .16 to .23 and from .16 to .20 for condition 1 and 3, respectively. Nevertheless, when the CRT-3 was administrated in the middle of the sequence (condition 2), the correlations were small and not statistically significant (correlations ranged from .10 to .13).

**Table 7:** Correlations between CRT scores and response time.

	Observed Correlations			Corrected Correlations		
	RT-C1	RT-C2	RT-C3	RT-C1	RT-C2	RT-C3
*Total Sample*						
CRT-13	.23**	.13	.20**	.29**	.17*	.25**
CRT-3	.16*	.10	.16*	.27**	.16*	.27*
CRT-10	.23**	.10	.18*	.31**	.13	.23**
*Men*						
CRT-13	.01	-.03	-.06	.01	-.04	-.07
CRT-3	-.02	.12	.02	-.03	.19	.04
CRT-10	.05	-.06	-.07	.06	-.08	-.10
*Women*						
CRT-13	.34**	.20*	.39**	.43**	.25**	.49**
CRT-3	.27**	.10	.28**	.46**	.17*	.48**
CRT-10	.33**	.16	.33**	.43**	.21**	.44**

*Note*. RT-C1 = response time of CRT in condition 1; RT-C2 = response time of CRT in condition 2; RT-C3 = response time of CRT in condition 3. CRT-13 = compound of CRT-3 [17] and CRT-10 [60].

*p ≤ .05

**p ≤ .01.

Similar results were found for women. When CRT-3 items were administrated at the beginning (condition 1) and at the end (condition 3) of the test, positive and statistically significant correlations were found (from .27 to .34 in condition 1 and .28 to .39 in condition 3), and small and not significant correlations were found when the CRT-3 was administrated in the middle of the sequence (condition 2; from .10 to .20). However, no relationship was found for men in any of the experimental conditions (from -.02 to .05 in condition 1, from -.06 to .12 in condition 2, and from -.07 to .02 in condition 3).

## Discussion

The main purpose of this study was to expand the literature on the determinants of CRT scores by exploring the effects that items sequence has on (1) CRT scores, (2) response time, (3) the relationship between CRT score and response time, (4) CRT scores, response time and the relationship between both variables based on the sex, and (5) the sex differences on CRT scores and response time.

The findings contribute to the CR literature in several ways. The first contribution of this research has been to evidence that items sequence has effects on CRT scores. The results showed that administrating the CRT-3 [6,17] in the middle or at the end of the CRT-10 items [60], the performance on CRT-13 and on CRT-3 improves significantly. Moreover, the results also showed that this effect is more pronounced on CRT-3 and that the items sequence does not have effects on CRT-10 performance. Hence, these findings suggest that CRT-3 items could be more affected by the sequence while CRT-10 items could be more robust against its effects. As previous researchers have displayed, these findings could be due to people noticing the items developed by Frederick [17] trickier than the items developed by other authors [16,64,65]. Consequently, administrating the CRT-3 items in the middle or at the end of the sequence, the performance on this test might improve taking the first items as training items.

The second contribution of this research has been to show that items sequence has no effects on CRT-13 response time. Nevertheless, the results suggest that participants spend more time answering the first items of the sequence. Hence, these findings also support that the first items could be used as training items, through which, participants become familiar with the mechanic of response. When participants are confronted to the CRT items for the first time, they could spend more time deliberating the first items due to the lack of familiarity with them. Consequently, the solution of the first items could trigger the automation of answers by means of consistent experience solving them. Thus, participants would win speed answering the last items.

The third contribution has been to prove that items sequence has effects on the relationship between CRT scores and response time. The results reported a positive relationship between CRT scores and response time when CRT-3 items were located at the beginning and at the end of the test. However, when the CRT-3 is administrated in the middle of the sequence, no relationship was found between variables. Hence, as previous studies, these findings suggest that higher scores on CRT could be associated for spending more time deliberating answers [46–48], but surprisingly when we get to maximize the CRT-3 performance, the response time could not determine the CRT scores.

Regarding to sex variable, the results have shown that items sequence has not the same effects on CRT scores, CRT response time, and on the relationship between these variables of women and men. These are, in fact, the fourth, fifth, and sixth contributions of this study. Concerning to CRT scores, findings showed that both women and men improved their CRT-3 scores administrating these items in the middle of CRT-10. In addition, women also improved their CRT-13 and CRT-10 scores. However, men´s performance on CRT-13 and CRT-10 were not affected by items sequence. Regarding to response time, results reported that items sequence had no effects on time that women need to answer the CRT items, but it had effects on men´s response time. Men spend more time answering the items that are administrated at the beginning of the sequence. Concerning the relationship between CRT scores and response times, results found a null correlation between these variables for men, independently of items sequence, but they found a positive correlation for women when CRT-3 are located at the beginning or at the end of the sequence.

Furthermore, as previous studies have shown (see, [34,65,66], for instance), this study also found that men systematically scored higher than women on CRT. The differences were higher on CRT-13, suggesting that the number of items could moderate the sex differences on CRT scores. Moreover, the differences were found independently from the items sequence administrated, which means that the items sequence does not moderate the sex differences on CRT scores. These are the seventh and eighth contributions of the current study.

Finally, results also reported that there were no differences between men and women in time responses regarding to items sequence. Therefore, the nineth contribution has been to prove that items sequence does not moderate the sex differences to the response time.

### Implications for the research and practice

The findings reported in this study suggest some implications for researchers and practitioners of all fields where the CR tests would be useful.

The results invite to practitioners and researchers to take in account the items sequence that CRT is administrated, always using the same sequence particularly when the CRT is applied with the purpose to make decisions that involves people (e.g., in personnel selection decisions). The findings also suggest that practitioners and researchers should not compare the CR level of people when the items sequence is not the same, due to this fact that it could bias their judgments or make erroneous inferences. Nevertheless, when the CRT-10 items are used alone as CR measure, the before issues must not be considered due to these items control robustly the effects of items sequence. Consequently, the results suggest the preferential use of the CRT-10 instead of the CRT-3. This is also justified in the great amount of variance that both measures shared. Previous studies have shown that CRT-10 and CRT-3 are highly correlated [41,65,67–69]. For this sample, the theoretical correlation (observed correlation corrected by measurement error in both measures) between CRT-10 and CRT-3 was .81, indicating that both tests are assessing the same construct, and consequently, one can be substituted for the other.

### Limitations and suggestions for future research

The current research has some limitations that should be noticed by readers. Despite that the sample size is large, the first limitation is that men and women sample is not balanced, being the sample size of men quite less than women. This fact implies that the men´s results are more affected by sample error and new studies should be carry out for replicating these findings. In addition, this sample was composed by adults of Western European Country, so new replications should be developed using other samples and countries.

Likewise, future research should extend this study by analysing the effects of items sequence has on CRT scores and response time controlling the possible effects on other test´s features, like the format of CRT (i.e., computer vs. paper and pencil; [70–72]).

## Conclusion

In summary, this paper presents an experimental study on the effects that items sequence of the CRT has on scores, response time, the relationship between both variables, and the sex differences on CRT scores and response time. The results showed that a manipulation of the items sequence had effects on CRT scores, but not had significantly effects on time spending response items. Although results suggest that the first items of the sequence could be working as training items. In addition, a positive relationship between CRT scores and response time was also found, except when the scores were maximized where no relationship was found. Finally, the findings suggested that the effects that items sequence has on these variables might be different in men and women.
